# Hybrid Granularities Transformer for Fine-Grained Image Recognition

**DOI:** 10.3390/e25040601

**Published:** 2023-04-01

**Authors:** Ying Yu, Jinghui Wang

**Affiliations:** School of Software, East China Jiaotong University, Nanchang 330013, China

**Keywords:** consistency feature, data enhancement, fine-grained image recognition, vision transformer

## Abstract

Many current approaches for image classification concentrate solely on the most prominent features within an image, but in fine-grained image recognition, even subtle features can play a significant role in model classification. In addition, the large variations in the same class and small differences between different categories that are unique to fine-grained image recognition pose a great challenge for the model to extract discriminative features between different categories. Therefore, we aim to present two lightweight modules to help the network discover more detailed information in this paper. (1) Patches Hidden Integrator (PHI) module randomly selects patches from images and replaces them with patches from other images of the same class. It allows the network to glean diverse discriminative region information and prevent over-reliance on a single feature, which can lead to misclassification. Additionally, it does not increase the training time. (2) Consistency Feature Learning (CFL) aggregates patch tokens from the last layer, mining local feature information and fusing it with the class token for classification. CFL also utilizes inconsistency loss to force the network to learn common features in both tokens, thereby guiding the network to focus on salient regions. We conducted experiments on three datasets, CUB-200-2011, Stanford Dogs, and Oxford 102 Flowers. We achieved experimental results of 91.6%, 92.7%, and 99.5%, respectively, achieving a competitive performance compared to other works.

## 1. Introduction

**Figure 1:**
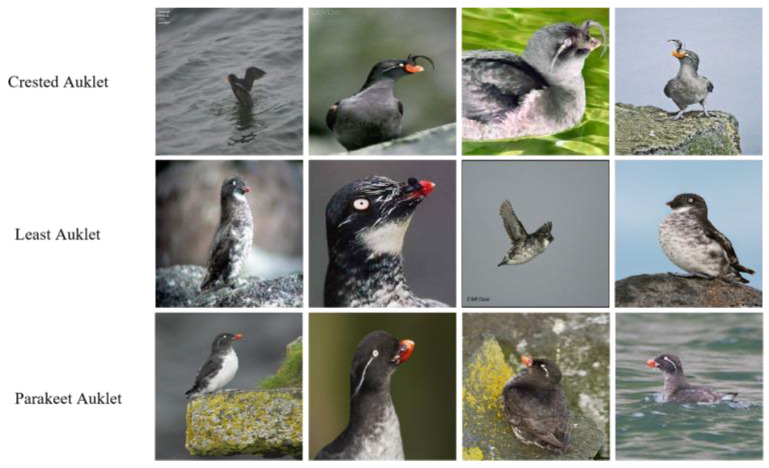
Different images of birds in the CUB dataset.

Fine-grained image recognition (FGIR) refers to the task of recognizing tiny differences between highly similar sub-categories within the same category, such as distinguishing between different species of birds or dogs [1]. It presents a greater difficulty compared to conventional image-classification tasks, because of its large variations in the same category and subtle differences among different categories. As shown in Figure 1, the birds in the image all belong to the broad category of Auklet, but the goal of FGIR is to identify their subcategories (e.g., Crested Auklet, Least Auklet, and Parakeet Auklet). In addition, issues such as shooting angles and target poses will pose another challenge for FGIR. However, since it has been applied in the medical and industrial fields, it has gained much attention.

In the field of fine-grained image classification, traditional classification methods are divided into strongly supervised and weakly supervised methods based on whether additional annotation information such as key points and bounding boxes is used. Strongly supervised classification methods use a large amount of manually annotated information, while weakly supervised classification methods only utilized image-level labels. In early strongly supervised methods, Zhang et al. [2] used a selective search algorithm to search for candidate boxes containing objects, added geometric constraints to the candidate boxes based on strong supervised annotation information, extracted feature information from different parts, and fused them to complete image classification, due to the need to generate a large number of candidate boxes and the consequent increase in training time.

Wei et al. [3] proposed a novel model as an alternative. They used a fully convolutional neural network (CNN) to obtain feature vectors for the whole object, the head, and the torso. By combining these three feature vectors using global max pooling and average pooling, they finally obtained a classification vector that could be used for prediction. Their model effectively aggregated features from different parts and suppressed interference from complex backgrounds. Steve et al. [4] utilized key point information to extract the local features of the target. Additionally, they further fused features from different levels to address the impact of varying object poses on the classification results. Di et al. [5] utilized a valve linkage function to connect various sub-networks, improving object localization and enhancing the network’s classification capabilities. Since strongly supervised methods require additional manual annotation, they can be time-consuming and resource-intensive. Furthermore, annotating objects requires a certain level of expertise, often necessitating the involvement of specialists, which can make obtaining annotation information challenging.

As deep learning continues to advance, increasingly robust convolution-based backbone networks have been proposed in the field of computer vision. In parallel, the supervised signals used for training the models have shifted toward image-level labels. Lin et al. [6] first introduced the bilinear network, which utilizes two backbone networks with shared weights to extract features from images and generate two feature vectors with the same dimension. These two vectors are then fused and passed through a fully connected layer for final classification. Fu et al. [7] proposed a multi-branch attention CNN, which used attention to crop images and identify discriminative features at different scales. Based on the previous work, Zhang et al. [8] utilized the maximum connected region of the feature maps to crop the target and fused the parts’ features from multiple branches, discovering more regional information. As the CNN model focuses on different regions of the target at different levels, Du et al. [9] proposed a multi-granularity progressive training model to address this issue. They proposed shuffling the image into different patches and using a progressive training approach, which can improve the robustness of the model. It can help shallow neural networks to mine edge details, while deeper networks can discover more semantic information. Hu et al. [10] proposed to crop and erase the attention maps in order to better locate the target and discover more discriminative regions. Additionally, they applied bilinear attention pooling to the feature maps and attention maps to obtain more accurate feature representations. In FGIR, the classification ability of the model was affected to some extent due to the interference of complex backgrounds. Rao et al. [11] proposed a causal reasoning counterfactual attention learning approach, which discovered effective features through powerful supervised signals and effectively suppressed the influence of background. Due to the tiny differences between different categories and the inconsistency of the regions focused by each channel in CNN, Gao et al. [12] proposed a channel-interaction model. They utilized contrastive learning to achieve channel interaction by discovering the complementary relationship between different channels within a single image and the differential information between channels across different images. In addition, Zhuang et al. [13] proposed a paired interacting learning network that utilizes contrastive learning to effectively discover the differences and relationships between different image pairs, thus discovering more complementary features.

Recently, more and more scholars and researchers have been utilizing Vision Transformer [14] (ViT) for downstream computer vision tasks. This model architecture has also been applied to the domain of FGIR in some research endeavors. For example, He et al. [15] takes into account the importance of each patch token in each layer, by summing up the attention weights of the first eleven layers and selecting the tokens with higher weights based on the sorted order. These selected tokens are then fed into the last layer of the network for classification. Moreover, they proposed a novel contrastive loss to expand intra-class differences. To address the limitation of ViT in lacking multi-level features, Wang et al. [16] proposed a novel feature fusion transformer. They filtered the tokens for each layer to help the network fuse local and multi-level features. A lot of work has been carried out on traditional the use of CNN-based methods in object localization and cropping. However, models based on ViT for object localization and cropping in FGIR have not yet been realized. Therefore, Hu et al. [17] proposed a localization and recognition transformer. They utilized the self-attention weights of each layer in the transformer and multiplied all the layer weights to obtain the final attention map. The regions with higher weights in the attention map were used for object localization and cropping. The cropped images were then enlarged to the original size and re-input into the network for classification. The method of using attention to erase objects has been widely used, and Liu et al. [18] applied this method to ViT. They masked the most discriminative parts of the object based on attention weights, forcing the network to discover other discriminative information. In addition, they also established a knowledge set to guide the network to learn features belonging to each class.

However, these studies applied the ViT directly to FGIR without taking into account the unique characteristics of the network architecture. In addition, some of the existing works based on the ViT are two-stage models. Typically, they require images to be fed into the network multiple times for feature extraction. Due to the nature of the two-stage model, it inevitably increases the training time and the difficulty of model convergence. Therefore, to tackle the aforementioned problems of existing models, we propose a novel model: Hybrid Granularities Transformer (HGTrans). In HGTrans, we propose Patches Hidden Integrator (PHI) and Consistency Feature Learning (CFL). In summary, this work presents several significant contributions:

(1) We propose a single-stage model and it can be trained end-to-end with only text labels.
(2) We propose the Patches Hidden Integrator (PHI) module to force the model focus on some other regions that are still discriminative in an efficient way.
(3) We propose the Consistency Feature Learning (CFL) module, which aids decision-making by discovering detailed information in the patch tokens and introduces an inconsistency loss as a constraint.
(4) Our proposed HGTrans outperforms existing models and achieves state-of-the-art results on several mainstream datasets.

## 2. Related Work

Due to the increased difficulty and challenges compared to general image classification tasks, FGIR has garnered much attention in the computer vision field. In recent years, a growing number of models and methods have been proposed for FGIR. Du et al. [19] fed a pair of images of the same category into the network and extracted the feature maps at different stages of the network. Based on the comparison between the feature maps of the same category at different stages, they proposed a category-consistency constraint to supervise the network to learn the most discriminative features within a category. Their methods achieved good results on different datasets. Peng et al. [20] proposed a progressive erasing network, where they performed different degrees of erasing on an image and fed them into the network. With a similar approach to ours, they proposed a consistency loss to encourage the network to learn different features of the same class. Our approach differs from theirs in that we calculate the inconsistency between the class token and patch token. Additionally, our approach also considers how the loss function should be adjusted when their classification results are consistent. In FGIR, some existing works have achieved good performance by mining diverse features, among which Chen et al. [21] proposed a Region Confuse Mechanism to disrupt the layout of images and learn the relationship between image blocks by recombining them. Their method also made appropriate changes to the input images, but what sets us apart is that we also leverage the features in the same class. This not only improves the model’s classification ability but also enhances its robustness. Furthermore, existing methods also involve mixing different images, among which Li et al. [22] proposed an attribute mixing model. They proposed to mix the features of two images in a certain proportion to generate a new image, which not only enriches the training samples but also helps the network to explore the attributes of the same parent category. Since the object usually only occupies a portion of the image, the other regions are considered as the background. In ViT, these background regions cause a lot of redundant computation. Therefore, Zhang et al. [23] proposed an Object Discriminator transformer. They selected patches with smaller weights (i.e., the background regions) based on the self-attention weights activation map, and then replaced these background regions with patches from other images. This effectively avoids unnecessary computations. However, their method is a two-stage model and introduces additional computation when obtaining the self-attention weight activation map. In contrast, our method does not require any additional parameters or computations.

## 3. Method

**Figure 2:**
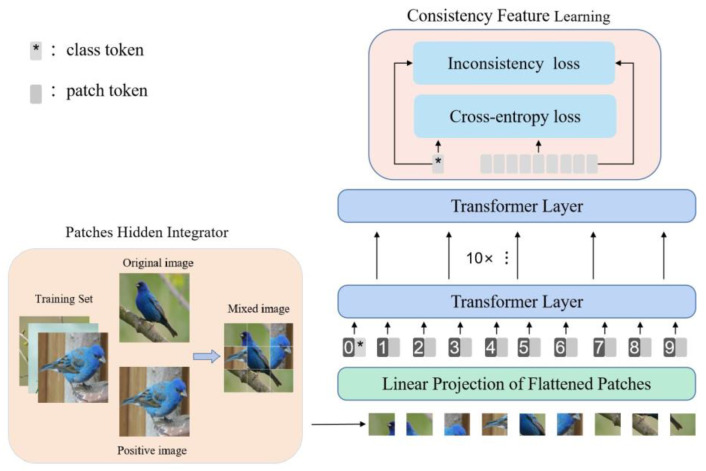
The framework of HGTrans.

**Figure 3:**
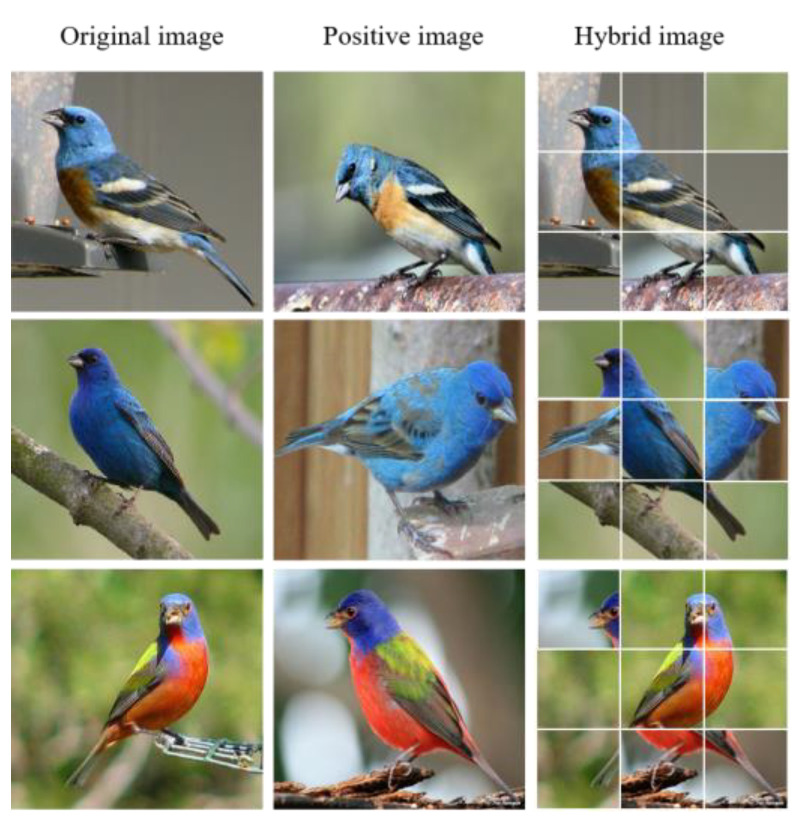
Diagram of the Patches Hidden Integrator module.

The architecture of HGTrans is shown in Figure 2. It contains the backbone network ViT, Patches Hidden Integrator (PHI), and Consistency Feature Learning (CFL). The different modules are marked with different colors in the diagram. In ViT, the image is initially divided into a series of patches, which are then fed into the Linear Projection. ViT utilizes a multi-head self-attention mechanism to discover the correlation among different image patches. In PHI, some patches will be randomly discarded. In this way, the model will focus on other discriminative regions, rather than just the most discriminative region. Furthermore, the discarded patches are supplemented by images of the same label (as shown in Figure 3), which encourages the network to find more consistent features in the same category. In CFL, we mine the local information hidden in the patch tokens and fuse it with the class token to assist in decision-making. On top of this, we also introduce an inconsistency loss function to supervise the network to learn consistency features. When the classification results of the class token and patch tokens are inconsistent, CFL calculates the inconsistency loss to measure the error between tokens in order to reduce ambiguity between them and better assist neural networks in classification. Before introducing the two proposed modules in detail, we first provide a brief overview of the datasets.

### 3.1. Datasets

Our experiments were conducted on three different datasets as follows. The CUB-200-2011 [24] dataset consists of a collection of 200 different bird classes and a total of 11,788 images, of which 5994 are used for training and 5794 are used for testing. Stanford Dogs [25] has 11,788 pictures, including 120 different dog species. Oxford 102 Flowers [26] was released in 2008, and it consists of 8189 images with 103 flower categories.

### 3.2. Patches Hidden Integrator (PHI)

Previous studies have proposed methods that require additional parameters or computational resources to extract discriminative information. However, this is not ideal for the high-complexity backbone Vision Transformer. In order to explore more features without increasing the training time, we propose the Patches Hidden Integrator.

For an image $x\in R^{H\times W\times C}$ with category y, it is first segmented into N p×p size patches before being fed into the network. H, W, C and p represent the image of height, width, channels, and the size of each patch, respectively. The patch sequence representation after image segmentation can be shown as follows:

(1) $$z_{x}=[x_{p}^{1},\;x_{p}^{2},\;x_{p}^{3},\;\ldots ,\;x_{p}^{N-1},\;x_{p}^{N}]$$

To encourage the model to focus on other important regions, PHI randomly selects a positive image $x_{pos}$ with category label y from the training set and divides it into L M×M patches.

(2) $$z_{x_{pos}}=[x_{M}^{1},\;x_{M}^{2},\;x_{M}^{3},\;\ldots ,\;x_{M}^{L-1},\;x_{M}^{L}]$$

Each patch in $x_{pos}$ has a probability P of being overlaid onto the original image x. The new image sequence representation after replacement can be shown as follows:

(3) $$z_{x_{new}}=[x_{p}^{1},\;x_{M}^{2},\;x_{M}^{3},\;x_{p}^{4},\;\ldots ,\;x_{M}^{N-1},\;x_{p}^{N}]$$

Extensive experiments have shown that the model performs best when P = 0.1 and M = 64. We will provide experimental details in the ablation study section. Due to the random replacement of some patches in each image x during the training process, the network avoids relying too much on a certain area (e.g., the wings or tail of a bird) for classification, forcing the network to discover other small key regions of the object (such as the eyes, beak, etc.). Additionally, since PHI blends the two images at a certain granularity during training, the network can discover additional features within a category. In general, PHI helps the model focus on secondary discriminative information in the image, while also discovering common features between different images of the same category, without adding extra computational overhead. In the training phase, premature replacement of patches can affect the classification ability of the network. Therefore, to avoid the negative effects of replacing patches during the early training process, we introduced PHI when some epochs had been trained (i.e., the PHI module was introduced only after 15 epochs of training were completed, during the entire training process of 100 epochs). As the network already has the ability to discover multi-discriminatory regions after training, PHI was not introduced during the testing phase. The PHI module was only used during training.

### 3.3. Consistency Feature Learning (CFL)

In the structure of ViT, a pre-defined class token is used to learn the classification information of all categories, and the global information contained therein is crucial for image classification. However, in the patch tokens, each patch represents a part of the image, which naturally contains more local details. However, previous works have typically ignored the detailed information in the patch tokens. In CFL, we explore the information in patch tokens for classification and fuse it with the classification results of the class token to assist in decision-making. Specifically, we concatenate the patch tokens output from the last layer to obtain a feature vector $F_{patch}$.

Following ViT, we classify the class token and $F_{patch}$ by two different classifiers. This helps to explore the neglected features in the patch tokens. When $x_{class}$ and $F_{patch}$ go through a classifier and the max function is applied to obtain the index (i.e., the represented class number) with the highest probability, the final classification result is obtained.

(4) $$y_{class}=\mathrm{Max}(Classifier(x_{class}))$$

(5) $$y_{patch}=\mathrm{Max}(Classifier(F_{patch}))$$

where $x_{class}$ is class token, $y_{class}$ and $y_{patch}$ are the classification results. Furthermore, we aim to eliminate the inconsistency of the classification results for both of them and force the network to learn consistent features between them, and we introduce an inconsistency loss. When they have the same classification result for the same image, we do not count the loss between them. Otherwise, we calculate the distance between them as the supervisory signal.

(6) $$Loss_{inconsis}=\left\{\begin{array}{l} \sum_{i=1}^{B}{\left|{\left\|x_{class}\right\|}_{F}-{\left\|F_{patch}\right\|}_{F}\right|}^{2},y_{class}\neq y_{patch}\\ \;\;\;\;0\;\;\;\;,y_{class}=y_{patch} \end{array}\right.$$

where B is the batch size, and ${\left\|\cdot \right\|}_{F}$ is the Frobenius norm. Therefore, the total loss of the model during the training phase is

(7) $$L_{CE}(y_{class},y)=-\sum_{i=1}^{B}y_{class}^{i}\times log(y^{i})$$

(8) $$L_{CE}(y_{patch},y)=-\sum_{i=1}^{B}y_{patch}^{i}\times log(y^{i})$$

(9) $$L{\mathrm{oss}}_{total}=L_{CE}(y_{class},y)+L_{CE}(y_{patch},y)+\alpha Loss_{inconsis}(x_{class},F_{patch})$$

where $L_{CE}()$ is the cross-entropy loss and y is the true label. Moreover, we set a weight parameter α for the inconsistency loss to be adjusted to better encourage the network to mine consistency features. The performance of the model also varies with different values of α. When α=3, the model achieves the best performance. We will provide specific details in the experimental section.

## 4. Experiments

### 4.1. Implementation Details

In order to be consistent with prior work, we used ViT-B_16 as our framework network and loaded the imagenet21k pre-trained weights. We conducted experiments on three mainstream datasets. To increase the diversity of the training data, we enlarged the image to 550 × 550 and randomly cropped it to 448 × 448. We also applied random flipping to the training images. At the testing stage, to improve the efficiency of image prediction, we only performed center cropping on the test images. We employed the SGD as our model optimizer. The cosine annealing algorithm was used to adapt the learning rate, and the initial learning rate was 0.001. The parameter M, which denotes the size of each patch, was set to 64, and the probability P was 0.1. The parameter α for the inconsistent loss was set to 3, and the batch size was 8. All experiments were conducted on the PyTorch.

### 4.2. Evaluation Indicators

The evaluation metric commonly used in FGIR is classification accuracy. Consistently with previous works, we make predictions on the test set images, and record the number of images that are classified correctly by the model. We calculate the ratio of the number of correctly classified images to the total number of images in the test set as the accuracy of our model. The formula is defined as follows:

(10) $$\mathrm{Accuracy}=\frac{\left|Correct\right|}{\left|\mathrm{Total}\right|}$$

### 4.3. Comparison with the State-of-the-Art

We conducted comparative experiments with different methods on three datasets, and the outcomes are displayed in Table 1 and Table 2. In Table 1, PMG-V2 gradually mines features at different granularities through progressive training. It achieved the best performance of 90.0% in CNN-based work on the CUB dataset. In comparison, our method HGTrans outperformed it by 1.6%. Additionally, our method HGTrans demonstrated a 0.9% improvement over the traditional ViT and outperformed the two-stage models RAMS and TPSKG by 0.3%, achieving the highest performance. For the Stanford Dog dataset, even the basic ViT achieved good performance, surpassing most CNN-based methods. However, our method HGTrans can discover more discriminative features compared to it, and we still achieved a 0.7% improvement over it. The experimental results for the Oxford 102 Flowers dataset are shown in Table 2. For this dataset, existing methods have already achieved remarkable performance. This indicates that both CNN-based and ViT-based methods have achieved the expected performance level. Our method HGTrans not only outperformed ViT by 0.2% but also achieved the same result as TPSKG. Additionally, our method is a single-stage model with lower training complexity, while TPSKG is a two-stage model, which further demonstrates the effectiveness of our proposed method.

### 4.4. Ablation Studies

To confirm the validity of Patches Hidden Integrator (PHI) and Consistency Feature Learning (CFL) in HGTrans, we conducted ablation experiments on the CUB-200-2011 dataset. The specific experimental outcomes are presented in Table 3. In Table 3, we introduce PHI and CFL to ViT separately to observe the effect of each module on the experimental results. When we introduced PHI and CFL, the model performance improved by 0.5% and 0.6%, respectively, relative to the most basic ViT. When they are used together in combination, the model performance reaches a maximum of 91.6% at this point. The experimental results show that both of our proposed modules are effective.

**Figure 4:**
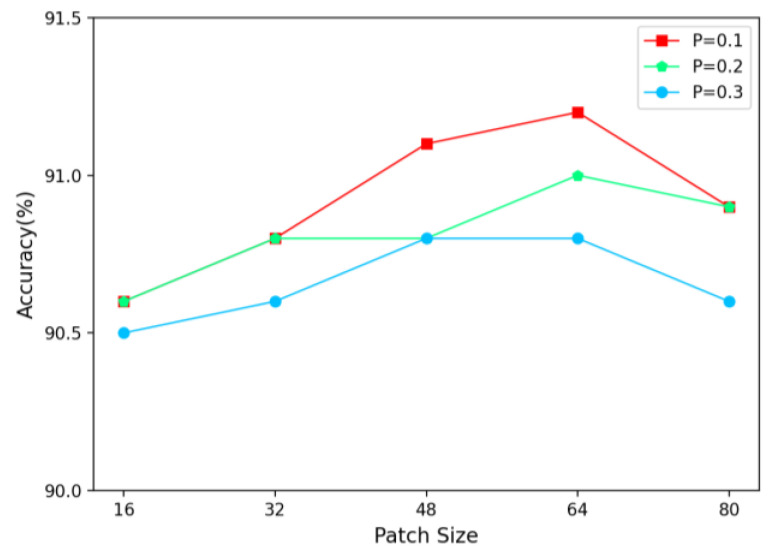
Effect of different values of (M, P ) on PHI module.

In addition, we also evaluated the effect of the values of different parameters (M, P) on the experimental results. In Figure 4, the horizontal coordinate represents the value of the parameter M (the size of each patch), the vertical coordinate is the accuracy rate, and the different colored lines indicate the size of the probability parameter P. As M increases in a certain range, the area covered by each patch on the original image also becomes larger, allowing the network to better discover additional discriminative regions, thus gradually improving its performance. When M is low, patches may not cover important areas well, and may even negatively affect the network’s performance. When M remains constant, a larger P value results in more patches, and more areas being covered up. Only a proper number and size of patches can strengthen the classification ability of the network, as too many or too few patches can have negative impacts. Therefore, by the experimental results, the P value is set to 0.1 and M is 64.

Different values of the parameter α have an impact on the experimental results, so we also conducted ablation experiments. In Table 4, the experimental results indicate that as the value of the parameter α increases from 1 to 3, the inconsistency loss supervised network learns some common features, which is beneficial for the network’s classification. However, as α continues to increase, the overly large weight causes the network to learn some invalid features, resulting in a gradual decrease in the network’s classification performance.

To make a more intuitive comparison, we also recorded the training time for different methods for one epoch. As shown in Table 5, ViT has the shortest training time, taking only 6 min and 14 s, followed by our method at 6 min and 25 s, while the two-stage RAMS model takes the longest time at 16 min and 5 s. Since our method is a single-stage model, it only increases the training time by 11 s. Compared to RAMS, we not only reduced the training time but also improved the accuracy, which indirectly proves the effectiveness of our method.

### 4.5. Visualization

**Figure 5:**
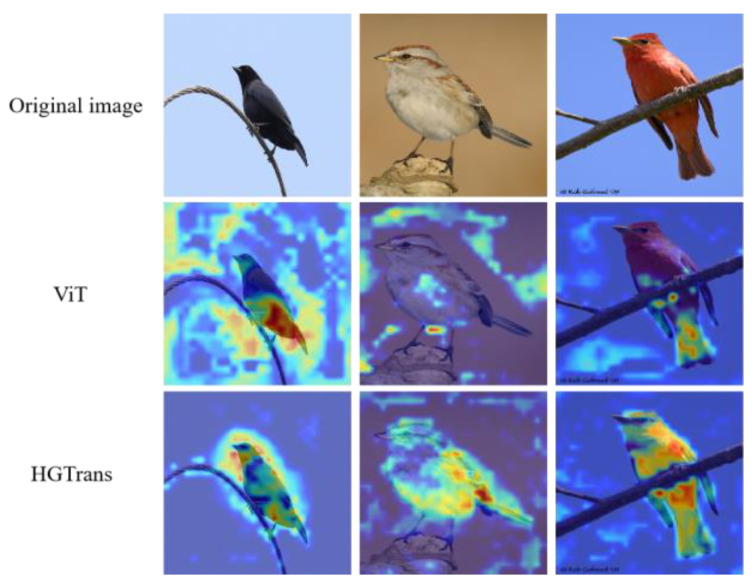
Visualization results of HGTrans on the CUB dataset.

In this section, we visualized the results of our model in Figure 5. Through attention activation maps, we can see that traditional ViT tends to focus more on specific regions of the target, which is the reason for its unsatisfactory performance. With the help of the two modules (PHI and CFL), our model can pay more attention to the overall target and discover more classification features. Therefore, from the visualization results, the two proposed modules PHI and CFL have played their expected roles.

## 5. Conclusions

In this paper, we proposed two lightweight modules, Patches Hidden Integrator (PHI) and Consistency Feature Learning (CFL), to encourage the model to mine more discriminative regions and detailed information. It helps improve the model’s classification capability and robustness. We also experimented on CUB-200-2011, Stanford Dogs, and Oxford 102 Flowers datasets. The experimental results showed that our method achieved performance of 91.6%, 92.7%, and 99.5%, respectively, with these datasets. We verified the effectiveness of each module in ablation experiments. We hope to expand the application of fine-grained image recognition to areas such as intelligent retail, plant pathology recognition, animal conservation, and more. In future work, we will design a new module to discover complementary relationships between different layers and improve the performance of the network.

## Figures and Tables

**Table 1:** Experimental results of CUB and Dog.

Method	CUB (Accuracy)	Dog (Accuracy)
RA-CNN [7]	85.3	87.3
MAMC [27]	86.5	85.2
SEF [28]	87.3	88.8
Cross-X [29]	87.7	88.9
FDL [30]	89.1	84.9
FBSD [31]	89.8	89.4
API-NET [13]	90.0	90.3
PMG-V2 [19]	90.0	90.7
ViT [14]	90.7	92.0
RAMS [17]	91.3	92.4
TPSKG [18]	91.3	92.5
**HGTrans**	**91.6**	**92.7**

**Table 2:** Experimental results of Flower.

Method	Flower (Accuracy)
PBC [32]	96.1
PC-CNN [33]	93.6
BiM-PMA [34]	97.4
Grafit [35]	99.1
BiT m [36]	99.3
ViT [14]	99.3
TPSKG [18]	99.5
**HGTrans**	**99.5**

**Table 3:** Ablation studies on different modules.

ViT_B_16	PHI	CFL	Accuracy (%)
**✓**			90.7
**✓**	**✓**		91.2
**✓**		**✓**	91.3
**✓**	**✓**	**✓**	91.6

**Table 4:** Experimental results for different parameters.

Method	α	Accuracy (%)
HGTrans	1	90.9
HGTrans	2	91.3
HGTrans	3	91.6
HGTrans	4	91.4

**Table 5:** Training time for different methods.

Method	Time (Min)
ViT	6:14
HGTrans	6:25
RAMS	16:05

## Data Availability

Not applicable.
